# An exploration of happiness, anxiety symptoms, and depressive symptoms among older adults during the coronavirus pandemic

**DOI:** 10.3389/fpsyg.2023.1117177

**Published:** 2023-03-30

**Authors:** Melanie M. Y. Serrao Hill, Nancy Hauck, Jeremy B. Yorgason, Caroline Bown, Kortney Tankersley

**Affiliations:** ^1^Optum Inc., Eden Prairie, MN, United States; ^2^Department of School of Family Life, Brigham Young University, Provo, UT, United States; ^3^Department of Community and Global Engagement, Dixie State University, St. George, UT, United States

**Keywords:** COVID-19, depression, anxiety, resilience, well-being, later life

## Abstract

The COVID-19 pandemic has affected millions of people worldwide. Because of the challenges associated with the pandemic, universal levels of happiness have likely depleted. We know little about how those with prior existing mental health concerns have responded to the pandemic. Using cross-sectional (study 1; *N* = 1,366) and longitudinal (study 2; *N* = 262) data, we utilized a stress and resilience perspective to explore mental health symptoms and happiness among older adults before and after the declaration of the pandemic. Results for both studies indicated higher levels of depression and anxiety symptoms predicted lower levels of happiness; however, for those who indicated higher levels of mental health symptoms, post-pandemic declaration happiness levels were higher than pre-pandemic happiness levels. Findings suggest that resilience may be learned throughout a lifetime, and that experiences from prior stressors may show benefits in responding to future ones, even among vulnerable populations.

## Introduction

The coronavirus disease 2019 (COVID-19) pandemic has affected millions of people worldwide, with severe illness, hospitalization, and death rates highest among older adults (Center for Disease Control [CDC], 2021). Additionally, the pandemic has impacted individuals mentally and emotionally (Li et al., 2020; Serafini et al., 2020; Xiao et al., 2020; Casali et al., 2021). The psychological effects of an unprecedented event like the pandemic are far from being fully explained by research— as researchers are still investigating the pandemic’s repercussions on mental health and happiness (Pfefferbaum and North, 2020).

Indeed, the pandemic may have exacerbated previously experienced challenges. For example, Serafini et al. (2020) found that many older adults experienced negative psychological well-being, such as anxiety and disabling feelings of loneliness, during the pandemic. Further, various daily stressors have been found to predict mood (Almeida, 2005; Almeida et al., 2009), indicating that when multiple stressors compound on one another in a “pile-up” fashion, negative mood may be intensified on a daily basis and over time. As such, older adults exhibiting anxiety and depressive symptoms may also report lower levels of happiness, and due to stress pile-up, this relationship may be exacerbated with the COVID-19 pandemic (Wang et al., 2020). A stress perspective might suggest that older adults would be more impacted than younger adults by the compounding physical and emotional challenges brought on by COVID-19 (Colenda et al., 2020).

On the other hand, previous research on resilience in later life suggests that older adults may have an arsenal of attributes to buffer negative outcomes (MacLeod et al., 2016). Research conducted since the COVID-19 pandemic has supported this notion, showing that older adults have been some of the most resilient during this time (Fuller and Huseth-Zosel, 2021). Many have adjusted positively to social isolation, sheltering in place, and other hygienic precautions, likely due to their understanding of the virus’s threat. Such proactive coping may strengthen resilience (Pearman et al., 2021). In fact, older age during the pandemic has been linked to lower depression and anxiety symptoms among an Italian sample (Rossi et al., 2020). Likewise, older adults have grown in their knowledge of technology, using these resources to stay connected to loved ones, which has helped them feel less isolated than before the pandemic (Colenda et al., 2020). While the impact of the pandemic on older adults continues to unfold, more work is needed to understand the biological, psychological, and social outcomes of this global phenomenon.

Although some older adults show evidence of stress and others show evidence of resilience, more research is needed to understand the complexities of why people have responded in different ways. We address this question by exploring how associations between anxiety and depressive symptoms and happiness might have changed during the pandemic. Following the stress perspective, anxiety and depressive symptoms combined with the pandemic could have a pile-up impact on happiness. Alternatively, from a resilience perspective, the pandemic might have lessened the impact of mental health challenges in relation to happiness. Using cross-sectional (study 1) and longitudinal (study 2) data, the purpose of the current study was examine how anxiety and depressive symptoms were related to happiness among older adults, along with how the COVID-19 pandemic moderated these relationships.

Researchers have discussed many determinants of well-being (see George, 2010) with one important effect on happiness being mental health. In later life, depressive symptoms (Kroemeke and Gruszczynska, 2016; Luchesi et al., 2018) and anxiety symptoms (i.e., worry; Fakouri and Lyon, 2005) have been linked with lower life satisfaction and negative affect. In fact, some researchers have found that mental health symptoms may be even more predictive of lower life satisfaction than physical health symptoms (Ghubach et al., 2010). As such, the first aim of the current study was to examine the relationship between anxiety and depressive symptoms and happiness in later life.

Although the stress perspective is supported by studies of older adult responses to disasters, researchers have also found evidence of resilience and adaptation to stressors brought on by challenging events (Lim et al., 2015; Alpass et al., 2016). Resilience in later life is often portrayed as combining biological, psychological, social, and spiritual capacities to adjust to adversity (Tay and Lim, 2020). Stress inoculation, which includes learned stress management coping skills, may also contribute to resilience as it has been linked with improved psychological health, self-efficacy, and happiness in older Iranian women with depression (Banisi, 2020). Such findings suggest that resilience may be a unique aspect of later life globally.

Recent research has supported ideas of resilience during the COVID-19 pandemic, with older adults demonstrating impressive subjective well-being and steady mental health (Chen, 2020; Fuller and Huseth-Zosel, 2021; Kwon, 2021). Perhaps the combination of chronological age, life wisdom, and prior experience coping with disasters have created a protective buffer for many older adults during the current pandemic (Miller and Brockie, 2015; An et al., 2019). In fact, previous research indicates a significant relationship between resilience and stressful life events among older adults, with resilience being associated with lower levels of depressive symptomology (Lim et al., 2015) and fewer negative emotional effects (Miller and Brockie, 2015). Therefore, as older adults demonstrate significant resilience, some scholars have suggested that they may be impacted less negatively by psychological challenges brought on by the COVID-19 pandemic (Vahia et al., 2020). Because literature is mixed in relation to how middle age and older adults cope with stress (Almeida, 2005; Almeida et al., 2009), and how they demonstrate resilience in difficult situations (Lim et al., 2015; Alpass et al., 2016), the second aim of the current study was exploratory in nature. We examined how the COVID-19 pandemic was related to happiness in later life.

Although the general older adult population may exhibit moments of resilience during difficult times like a pandemic, there may be subgroups who are at risk for experiencing negative outcomes during stressful events. Specifically, individuals who endure anxiety or depressive symptoms may find that the COVID-19 pandemic has exacerbated previously experienced challenges and even brought about new concerns. Individuals living with mental illness reported being very concerned about service disruption, running out of medication, and social isolation during the COVID-19 pandemic (Costa et al., 2020). Further, in a study assessing 1,310 Spanish adults, individuals who reported fewer positive emotions and higher loneliness also reported greater distress during the pandemic (Losada-Baltar et al., 2021). As previous research has found that various daily stressors predict mood (Almeida, 2005; Almeida et al., 2009), perhaps over time, multiple stressors (e.g., mental health symptoms and a pandemic) compound in a “pile-up” fashion, therefore exacerbating negative outcomes (e.g., Wang et al., 2020). As such, the final aim of the current study was to examine whether the COVID-19 pandemic moderated the relationship between anxiety and depressive symptoms and happiness in later life.

### The present studies

The COVID-19 pandemic has placed significant strains on individuals throughout the world. Because of the challenges associated with the pandemic, universal levels of happiness have likely depleted. Although some have clearly been negatively affected by the pandemic, and many older adults have demonstrated resilience throughout, less is known about how those with prior existing mental health concerns responded to the pandemic.

The purpose of the current study was to explore the relationship between anxiety and depressive symptoms and happiness, while simultaneously examining whether the COVID-19 pandemic moderated this relationship. We tested associations cross-sectionally, using two samples of older adults: one group who took our survey prior to the COVID-19 pandemic declaration, and the other who took our survey after the declaration (study 1). Next, we tested the relationship longitudinally, using a sample of older adults who completed the survey prior to the COVID-19 pandemic declaration as well as after the declaration (study 2). Drawing on the stress and resilience literatures to exploring later life, we proposed the following hypotheses:

Hypothesis 1a: Depressive symptoms will be associated with lower levels of happiness.

Hypothesis 1b: Anxiety symptoms will be associated with lower levels of happiness.

Hypothesis 2a: Based on a stress “pile-up” approach, we hypothesized that survey responses during the COVID-19 pandemic would be associated with lower levels of happiness.

Hypothesis 2b: Based on a resilience approach, we hypothesized that survey responses during the COVID-19 pandemic would be associated with stable levels of happiness.

Hypothesis 3: Based on a stress pile-up approach, we hypothesized that time of survey completion (pre-pandemic vs. post-pandemic declaration) would moderate the association between mental health symptoms and happiness. Specifically, we hypothesized that higher levels of depression and anxiety symptoms during the COVID-19 pandemic would be associated with lower levels of happiness.

## Study 1

### Materials and methods

#### Sample and procedure

The sample for the current study was drawn from a larger community initiative, surveying residents of a county of approximately 180,000 people in a Western state in the United States of America between 17 November 2019 and 5 June 2020. With approval from a local university’s Institutional Review Board (IRB), participants were recruited and contacted through various means, including posted/distributed flyers, emails, third party/non-study personnel/professional recruiters, websites and social media sites, newspaper and local magazine ads, and announcements at public events. When contacted, potential participants were informed about the current study and asked to complete a survey. If individuals were willing, they were given a link to the survey. Upon accessing the Qualtrics survey, participants agreed to an informed consent before participation. Inclusion criteria to participate in the study included any adult (18+) who was a resident of the participating county. Participants were entered into a drawing for gift cards at local businesses. Total sample included 2,337 residents. For the current study, only those 50 and older (*N* = 1,366) were included in analyses.

Participants in the current study included 1,338 older (*M*_*age*_ = 68.0, *SD* = 8.91, range = 50−97) individuals (64.91% female, 35.09% male). The majority of adults were in a romantic relationship (i.e., in a committed relationship, married, remarried, living with life partner; 76.18%). Socioeconomic characteristics of the sample suggest a wide distribution of income with 41.53% having an average household income between $50,000 and $99,999, 22.90% between $10,000 and $49,999, 22.18% between $100,000 and $149,999, 11.94% making $150,000+, and 1.45% making under $10,000; however, participants were highly educated with 73.61% having a college degree and 67.67% were retired. Respondents had characteristics that were reflective of the area where data were collected (White = 95.22%, Latino = 1.57%, Native American = 1.27%, Asian = 0.97%, Polynesian = 0.22%).

#### Measures

##### Happiness

Happiness was measured using a one item question asking, “In general, I consider myself.” based on a Likert scale ranging from 1 (*an extremely unhappy person*) to 7 (*an extremely happy person*).

##### Anxiety and depression symptoms

Anxiety and depression symptoms were measured using the Patient Health Questionnaire-4 (PHQ-4) tested by Löwe et al. (2010). Participants were asked to answer four items in response to the question, “Over the last 2 weeks, how often have you been bothered by the following problems?” To assess anxiety symptoms, the first two items addressed “feeling nervous, anxious, or on edge” and “not being able to stop or control worrying.” Depression symptoms were examined with the last two items addressing “feeling down, depressed, or hopeless” and “little interest or pleasure in doing things.” The items were based on a Likert scale ranging from 0 (*not at all*) to 3 (*nearly every day*). Higher scores indicated greater anxiety and depression symptoms. Both measures showed adequate internal consistency and reliability (depression symptoms: α = 0.82; anxiety symptoms: α = 0.78).

##### Pre-pandemic and post-pandemic declaration

The larger study in which the analytic sample was taken from did not intend to take place during a worldwide pandemic. Data collection began prior to the onset of the COVID-19 pandemic. However, the researchers used the opportunity to examine whether the pandemic influenced our study’s main variables. To do so, a variable was created so that those who completed the survey prior to 12 March 2020 received a 0 (pre-pandemic), and those who completed the survey after 12 March 2020 received a 1 (post-pandemic declaration).

Variables related to pandemic stress, such as the fear of social isolation, loss of physical health, and financial hardship were measured in the larger study initiative in which the current study’s analytic dataset was drawn from. Because there is overlap between pandemic related variables and those measured in the larger study, we feel that there is a strong connection between our findings and the impact of the COVID-19 pandemic. However, the current study did not measure specific pandemic-related variables, like the fear of infection. We acknowledge retrospectively that the fear of infection may have produced cognitive effects with how individuals interacted with others (e.g., Federico et al., 2021). Further, a study by Schimmenti et al. (2020) found that fear of infection might be conceptualized as a multidimensional phenomenon generated by interactions among bodily, interpersonal, cognitive, and behavioral domains. However, in the US and particularly in the location where the study was carried out, the fear of being infected by others may not have been as prominent a predictor of COVID-19 stress experience as in other parts of the world.

##### Covariates

Models were adjusted for age, relationship status (0 = not in a relationship, 1 = in a relationship), gender (0 = male, 1 = female), education (0 = not a college grad, 1 = college grad), and work status (0 = not working, 1 = employed, 2 = retired).

#### Analysis plan

Using STATA software (StataCorp, 2017), descriptive statistics and correlations were examined for each of the main study variables (see Tables 1, 2). For study 1, using the whole analytic dataset (*n* = 1,366), depression and anxiety symptoms were modeled as predictors of happiness within a multivariate regression model, controlling for age, gender, marital status, education, and retirement status. To examine whether the COVID-19 pandemic moderated the associations of anxiety and depressive symptoms and happiness, we modeled the interaction of time of when the survey was taken (pre- or during COVID) with anxiety and depressive symptoms as predicting happiness.

**TABLE 1:** Descriptive statistics for study 1 with the total sample (*N* = 1,366).

	Total sample (*N* = 1,366)			Pre-COVID (*N* = 654/47.88%)			Post-COVID (*N* = 712/52.12%)		
**Variable**	**M/% (SD)**	**Range**	***N***	**M/% (SD)**	**Range**	***N***	**M/% (SD)**	**Range**	***N***
Age	67.81	50−95	1,352	68.71^a^	50−95	648	66.99^b^	50−92	704
Gender	0.65	0−1	1,359	0.67	0−1	650	0.64	0−1	709
Male	34.88%		474	33.08%		215	36.53%		259
Female	65.12%		885	66.92%		435	63.47%		450
Marital status	0.77	0−1	1,366	0.76	0−1	654	0.77	0−1	712
Not in relationship	23.50%		321	24.46%		160	22.61%		161
In relationship	76.50%		1,045	75.54%		494	77.39%		551
Education	0.74	0−1	1,364	0.75	0−1	654	0.73	0−1	710
Not college grad	25.81%		352	24.77%		162	26.76%		190
College grad	74.19%		1012	75.23%		492	73.24%		520
Employment status	1.62	0−2	1,363	1.63	0−2	652	1.62	0−2	711
Not working	5.63%		73	5.67%		37	5.06%		36
Employed	26.85%		366	25.31%		165	28.27%		201
Retired	67.79%		924	69.02%		450	66.67%		474
Depression	0.30	0−3	1,292	0.28	0−3	627	0.31	0−3	665
Anxiety	0.39	0−3	1,293	0.33^a^	0−3	627	0.44^b^	0−3	666
Happiness	6.11	1−7	1,285	6.10	1−7	619	6.12	1−7	666

All means did not differ except where noted with superscripts.

**TABLE 2:** Bivariate correlations for Study 1 with the full sample.

	1	2	3	4	5	6	7	8
1. Happiness	−							
2. Depression	−0.51***	−						
3. Anxiety	−0.37***	0.62***	−					
4. Taken survey	0.01	0.03	0.09***	−				
5. Marital status	0.12***	−0.09**	−0.01	0.02	−			
6. Age	0.09**	−0.17***	−0.22***	−0.10***	−0.13***	−		
7. Gender	−0.04	0.11***	0.19***	−0.03	−0.15***	−0.09**	−	
8. Education	0.01	−0.08**	−0.04	−0.02	0.04	−0.11***	−0.14***	−
9. Employment status	0.08**	−0.15***	−0.16***	−0.01	−0.02	0.47***	−0.09**	−0.01

*p < 0.05, **p < 0.01, ***p < 0.001.

### Results

#### Descriptive statistics

Descriptive statistics and bivariate correlations are presented in Tables 1, 2. On average, participants expressed fairly low levels of depression (Mean = 0.30, *SD* = 0.56) and anxiety symptoms (Mean = 0.39, *SD* = 0.63). Participants in the sample reported to be happy in general (Mean = 6.11, *SD* = 0.88, range = 1−7). As seen in Table 2, bivariate correlations between the independent and dependent variables were of the expected magnitude and in expected directions. Depression (*r* = −0.51, *p* < 0.001) and anxiety symptoms (*r* = −0.37, *p* < 0.001) were significantly and negatively associated with happiness, indicating more depression and anxiety symptoms were related to lower levels of happiness. Further, taking the survey during the pandemic (as opposed to before the pandemic) was slightly associated (Cohen, 1988) with higher levels of anxiety (*r* = 0.09, *p* < 0.01). Other notable correlations with happiness included age (*r* = −0.09, *p* < 0.01), marital status (*r* = 0.12, *p* < 0.001), and employment status (*r* = 0.08, *p* < 0.05), indicating that older age, being in a relationship, and being retired was slightly associated (Cohen, 1988) with higher levels of happiness.

#### Regression analyses

##### Depression symptoms

Ordinary least squares (OLS) regression was used to examine how depression symptoms predicted happiness in mid to later life (see Model 1 of Table 3). To address hypothesis 1a, depression symptoms were modeled as a predictor of happiness in mid to later life, controlling for age, gender, marital status, education, and retirement status. A significant overall model was found when predicting happiness (*F*(8, 1,252) = 56.50, *p* < 0.001). After controlling for all covariates, depression symptoms were significantly associated with happiness (β = −0.39, *p* < 0.001), with predictors accounting for 27% of the variance in happiness (*R*^2^ = 0.27).

**TABLE 3:** Regression coefficients of depression and anxiety symptoms predicting happiness in older adults, study 1.

	Model 1		Model 2	
**Variable**	**b/ß**	**SE/eta^2^**	**b/ß**	**SE/eta^2^**
**Depression** (*N* = 1,261)	−0.39***/−0.50	0.02/0.24	−0.47***/−0.60	0.03/0.25
Taken survey (pre- vs. post-COVID)	0.04/0.02	0.04/0.00	−0.05/−0.03	0.05/0.00
Marital status	0.17**/0.08	0.05/0.01	0.18***/0.09	0.05/0.01
Age	0.00/0.04	0.00/0.00	0.00/0.04	0.00/0.00
Gender	0.06/0.04	0.05/0.00	0.07/0.04	0.05/0.00
Education	−0.05/−0.03	0.05/0.00	−0.04/−0.02	0.05/0.00
Retirement status				
Employed	0.12/0.06	0.10/0.00	0.14/0.07	0.11/0.00
Retired	0.06/0.03	0.10/0.00	0.08/0.04	0.10/0.00
Interaction—during COVID			0.14***/0.15	0.04/0.01
*R* ^2^	0.27	−−	0.27	−−
**Anxiety** (*N* = 1,262)	−0.27***/−0.38	0.02/0.14	−0.68***/−0.48	0.06/0.15
Taken survey (pre- vs. post-COVID)	0.07/0.04	0.05/0.00	−0.01/−0.01	0.05/0.00
Marital status	0.26***/0.12	0.06/0.02	0.25***/0.12	0.06/0.02
Age	0.00/0.04	0.00/0.00	0.01/0.04	0.00/0.00
Gender	0.11*/0.06	0.05/0.00	0.11*/06	0.05/0.00
Education	0.01/0.01	0.05/0.00	0.02/01	0.05/0.00
Retirement status				
Employed	0.18^†^/0.09	0.11/0.00	0.19^†^/0.10	0.11/0.00
Retired	0.13/0.07	0.10/0.00	0.15/0.08	0.11/0.00
Interaction—during COVID			0.12**/0.14	0.08/0.01
*R* ^2^	0.16	−−	0.17	−−

^†^>0.05, *p < 0.05, **p < 0.01, ***p < 0.001. Controls included age (continuous), marital status (0 = not in a relationship, 1 = in a relationship), gender (0 = male, 1 = female), education (0 = not a college grad, 1 = college grad), and work status (0 = not working, 1 = employed, 2 = retired). ß = standardized regression coefficient. eta^2^ = partial eta squared calculated using “estat esize” as a post-estimation command in Stata.

##### Anxiety symptoms

Ordinary least squares regression was used to assess how anxiety symptoms predicted happiness in mid to later life (see Model 1 of Table 3). To address hypothesis 1b, anxiety symptoms were modeled as a predictor of happiness in mid to later life. A significant overall model was found when predicting happiness (*F*(8, 1,253) = 30.63, *p* < 0.001). After controlling for all covariates, higher anxiety symptoms were significantly associated with lower happiness (β = −0.27, *p* < 0.001), with predictors accounting for nearly 16% of the variance in happiness (*R*^2^ = 0.16).

##### COVID-19 pandemic

To address hypothesis 2, OLS regression models were used to assess whether the time when a participant took the survey was related to happiness in mid to later life (see Model 1 of Table 3). There was no significant relationship between when participants took the survey (pre-pandemic vs. post-pandemic declaration) and happiness in either of the models (depression or anxiety symptoms).

##### Moderation of depression and anxiety symptoms and pre-/post- pandemic declaration

###### Depression symptoms

**FIGURE 1:**
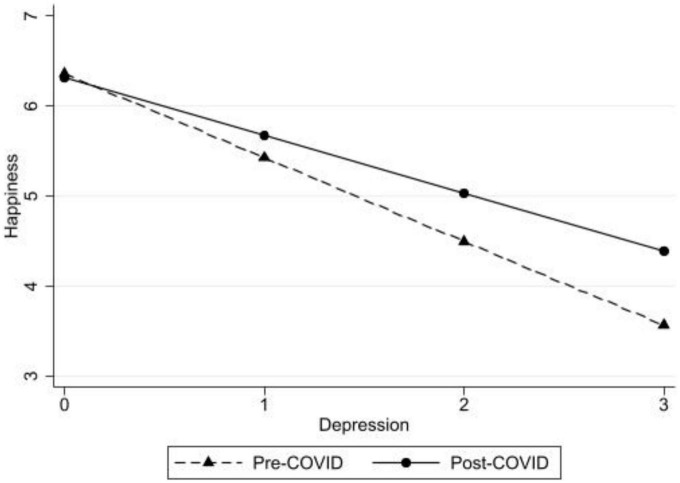
Depression symptoms predicting happiness pre- and post- the COVID-19 pandemic declaration with the full sample.

To address hypothesis 3a, if taking the survey pre- or post-pandemic declaration moderated the depression symptoms to happiness link among our sample, interaction effects were added to the regression models (see Model 2 of Table 3). Significant interaction effects were found between depression symptoms and survey time participation (pre-/post-pandemic declaration: β = 0.14, *p* < 0.001; see Figure 1 for a graphic representation of depression symptoms by pre-/post-pandemic declaration in predicting happiness). A simple slopes analysis indicated that higher depressive symptoms were significantly linked with lower happiness for both pre-and post-COVID-19 pandemic declaration respondents, yet the association was weaker post-pandemic declaration.

###### Anxiety symptoms

**FIGURE 2:**
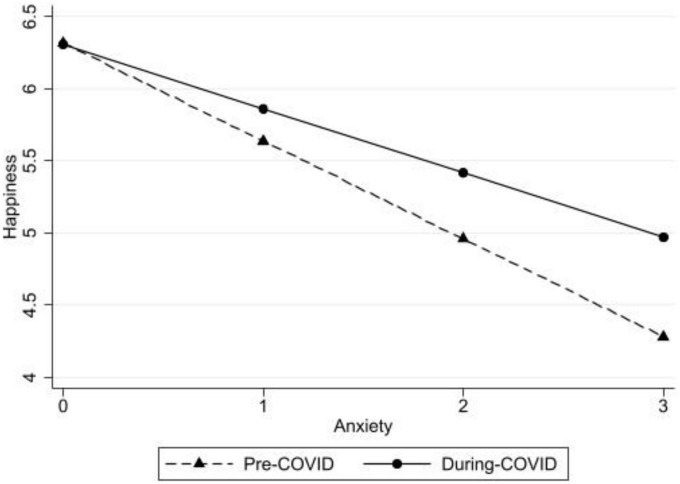
Anxiety symptoms predicting happiness pre- and post- the COVID-19 pandemic declaration with the full sample.

To address hypothesis 3b, if taking the survey pre- or during the COVID-19 pandemic moderated the anxiety symptoms to happiness link among our sample, interaction effects were added to the regression models (see Model 2 of Table 3). Significant interaction effects were found between anxiety symptoms and survey time participation (pre-/during pandemic: β = 0.23, *p* < 0.01; see Figure 2 for a graphic representation of anxiety symptoms by pre-/during pandemic in predicting happiness). A simple slopes analysis indicated that anxiety symptoms were significantly linked with happiness for both pre- and during COVID-19 pandemic respondents, yet the association was weaker during the pandemic.

### Discussion

Using data from 1,366 respondents to a community survey in a Western state of the US, we explored the relationship between happiness and anxiety and depressive symptoms, as well as how the pandemic might have moderated these mental health symptom’s associations with happiness. Consistent with our hypotheses and with prior literature, depression and anxiety symptoms were associated with lower reports of happiness in the sample (Perneger et al., 2004; Garaigordobil, 2015). Contrary to our expectations, the COVID-19 pandemic was not associated with reports of lower happiness. We also unexpectedly found that the pandemic had a buffering effect on associations between depression and anxiety and happiness. These results support a resilience perspective, that the negative association between anxiety and depressive symptoms and happiness was lessened during the pandemic among older adults. This finding is not completely unexpected, as other studies have suggested that older adults are demonstrating resilience during the COVID-19 pandemic (Fuller and Huseth-Zosel, 2021; Pearman et al., 2021). That said, results from the current analysis are limited in that they are based on cross sectional associations. The buffering impact of the pandemic on mental health symptom’s associations with happiness may differ when tracked across time among the same persons.

## Study 2

To assess if there was a longitudinal relationship of anxiety and depression symptoms on happiness, study 2 examined 262 older adults who participated in the same survey prior to and after the pandemic declaration. Although the sample for the current study was drawn from a larger community initiative created prior to the onset of the COVID-19 pandemic, the researchers for this study used the opportunity to better understand the study’s main variables in the context of a worldwide pandemic. The first wave of surveys was administered between November 17, 2019 and March 11, 2020, and the second wave of surveys were completed between March 12, 2020 and June 5, 2020.

### Materials and methods

#### Procedures and participants

County residents who participated in the larger study were asked whether they were willing to be contacted for a follow-up study. Initially, the follow-up study would have been conducted with more time between T1 and T2; however, with the onset of the COVID-19 pandemic, the researchers sent out the follow-up survey on March 12, 2020. Those who indicated that they were willing to participate in a follow-up study were sent an email with a link to the follow-up survey (which contained the same questions as the prior survey). Of the 502 emails sent to participants, 262 took part in the follow-up survey. Participants in the current study had a mean age of 69.17 (*SD* = 8.26, range = 50−95) with 71% identifying as female and 29% as male. The majority of older adults were in a relationship (75%), highly educated (78% college grad) and retired (73%).

##### Measures

Constructs were measured using the same items as Study 1. Happiness was measured using a one item question asking, “In general, I consider myself.” based on a Likert scale ranging from 1 (*an extremely unhappy person*) to 7 (*an extremely happy person*). Depression and anxiety symptoms were measured using four items from the Patient Health Questionnaire-4 (PHQ-4) tested by Löwe et al. (2010). Demographic information that was collected included age, relationship status (0 = not in a relationship, 1 = in a relationship), gender (0 = male, 1 = female), education (0 = not a college grad, 1 = college grad), and work status (0 = not working, 1 = employed, 2 = retired).

#### Analysis plan

For study 2, we examined data provided at two time points from the same respondents (pre- and post-pandemic declaration; *n* = 262). The mixed command in STATA was used to estimate multilevel models of happiness and the second time point predicted by time 1 anxiety and depressive symptoms, and the controls of age, gender, marital status, education, and retirement status. The mixed command utilized maximum likelihood estimation and adjusted for repeated measures of happiness across time.

### Results

#### Descriptive statistics

Descriptive statistics and bivariate correlations for Study 2 are presented in Tables 4, 5. At Time 1 (pre-pandemic declaration), participants expressed an average rating of.27 (*SD* = 0.48) on the depression symptoms scale and.34 (*SD* = 0.51) on the anxiety symptoms scale. Both scales ranged from 0 (*not at all*) to 3 (*nearly every day*) when the average of the items was considered. At Time 1 on the happiness scale, participants expressed an average of 6.11 (*SD* = 0.85) on a scale from 1 (*an extremely unhappy person*) to 7 (*an extremely happy person*). At Time 2, on average, participants expressed a rating of.32 (*SD* = 0.55) on the depression symptoms scale, 0.48 (*SD* = 0.69) on the anxiety symptoms scale, and 6.21 (*SD* = 0.75) on the happiness scale. As seen in Table 5, bivariate correlations between the independent and dependent variables were of the expected magnitude and in expected directions. Depression and anxiety symptoms at both time points were significantly and negatively associated with happiness at both time points (all *r*’s magnitude >−0.25, *p* < 0.001), indicating higher levels of depression and anxiety were related to lower levels of happiness both concurrently and over the pandemic.

**TABLE 4:** Descriptive statistics for study 2 (*N* = 262).

	Pre-COVID			Post-COVID		
**Variable**	**M/% (SD)**	**Range**	**N**	**M/% (SD)**	**Range**	**N**
Age	69.17^a^ (8.26)	50−95	261	69.65^b^ (8.55)	50−95	259
Gender	0.71	0−1	262	0.71	0−1	262
Male	29.39%		77	29.01%		76
Female	70.61%		185	70.99%		186
Marital status	0.75	0−1	262	0.76	0−1	261
Not in relationship	25.19%		66	24.14%		63
In relationship	74.81%		196	75.86%		198
Education	0.78	0−1	262	0.79	0−1	262
Not college grad	21.76%		57	20.99%		55
College grad	78.24%		205	79.01%		207
Employment status	1.68	0−2	262	1.68	0−2	262
Not working	5.34%		14	5.73%		15
Employed	21.37%		56	20.99%		55
Retired	73.28%		192	73.28%		192
Depression	0.27 (0.48)	0−3	256	0.32 (0.55)	0−3	250
Anxiety	0.34^a^ (0.51)	0−3	257	0.48^b^ (0.69)	0−3	252
Happiness	6.11^a^ (0.85)	1−7	254	6.21^b^ (0.75)	1−7	253

All demographic variables are taken from Time 1 of the study. All means did not differ except where noted with superscripts.

**TABLE 5:** Bivariate correlations for study 2 with the full sample.

	1	2	3	4	5	6	7	8	9	10
1. Happiness @ T1	−									
2. Happiness @ T2	0.56***	−								
3. Depression @ T1	−0.55***	−0.41***	−							
4. Depression @ T2	−0.38***	−0.50***	0.59***	−						
5. Anxiety @ T1	−0.45***	−0.29***	0.53***	0.48***	−					
6. Anxiety @ T2	−0.26***	−0.33***	0.25***	0.65***	0.45***	−				
7. Marital status	0.16**	0.12	−0.08	−0.14*	−0.15*	−0.11	−			
8. Age	−0.02	0.03	−0.09	−0.07	−0.18**	−0.11	−0.18**	−		
9. Gender	−0.14*	−0.07	0.07	0.07	0.17**	0.13	−0.21**	−0.07	–	
10. Education	0.04	0.03	−0.11	−0.14*	−0.05	0.00	0.08	−0.16**	−0.07	−
11. Employment status	0.10	0.09	−0.18**	−0.19**	−0.18**	−0.19**	−0.08	0.47***	−0.08	−0.04

All control variables were taken from T1 (pre-COVID). *p < 0.05, **p < 0.01, ***p < 0.001.

#### Multilevel models

##### Depression symptoms

Multilevel models using the mixed command in STATA were used to assess how depression symptoms predicted happiness in mid to later life for follow-up participants between pre- and post-pandemic declaration (see Model 1 of Table 4). To address hypothesis 1a, depression symptoms were modeled as a predictor of happiness in mid to later life. After controlling for all covariates, depression symptoms were significantly negatively associated with happiness (β = −0.47, *p* < 0.001).

##### Anxiety symptoms

Multilevel models were used to assess how anxiety symptoms predicted happiness in mid to later life for follow-up participants between pre- and post-pandemic declaration (see Model 1 of Table 4). To address hypothesis 1b, anxiety symptoms were modeled as a predictor of happiness in mid to later life. After controlling for all covariates, anxiety symptoms were significantly and negatively associated with happiness (β = −0.37, *p* < 0.001).

##### COVID-19 pandemic (measured *via* time)

To address hypothesis 2, Multilevel Models (MLM) models were used to assess whether time significantly predicted happiness in mid to later life (see Model 1 of Table 6). The variable time, which represented the time over which participants took the survey pre- and post-pandemic declaration, was significant at a trend level in both the depression (β = 0.06, *p* < 0.10) and anxiety symptoms (β = 0.06, *p* < 0.10) models in predicting happiness, indicating that happiness increased to some degree for these participants from pre-pandemic responses to during-pandemic responses.

**TABLE 6:** Multilevel regression coefficients of depression and anxiety predicting happiness in older adults, study 2.

	Model 1		Model 2	
**Variable**	**b/ß**	**SE**	**b**	**SE**
**Depression** (*N* = 255)	−0.38***/−0.47	0.04	−0.46***	0.05
Time (pre-vs. post-COVID)	0.09^†^/0.06	0.05	0.01	0.05
Marital status	0.19*/0.11	0.09	0.19*	0.09
Age	−0.00/0.02	0.01	0.00	0.01
Gender	−0.06/−0.04	0.08	−0.06	0.08
Education	−0.06/−0.03	0.09	−0.06	0.09
Retirement status				
Employed	0.43*/0.22	0.17	0.44*	0.17
Retired	0.29^†^/0.16	0.16	0.29^†^	0.16
Interaction depression × time			0.16**	0.05
**Anxiety** (*N* = 256)	−0.29***/−0.37	0.04	−0.38***	0.05
Time (pre- vs. post-COVID)	0.09^†^/0.06	0.05	−0.02	0.06
Marital status	0.13.07	0.10	0.13	0.10
Age	−0.00/−0.01	0.01	−0.00	0.01
Gender	−0.03/−0.02	0.09	0.03	0.09
Education	−0.02/−0.01	0.10	−0.02	0.10
Retirement status				
Employed	0.59*/0.30	0.19	0.60**	0.19
Retired	0.42*/0.23	0.18	0.43*	0.18
Interaction anxiety × time			0.17***	0.05

^†^>0.05, *p < 0.05, **p < 0.01, ***p < 0.001. Controls included age (continuous), marital status (0 = not in a relationship, 1 = in a relationship), gender (0 = male, 1 = female), education (0 = not a college grad, 1 = college grad), and work status (0 = not working, 1 = employed, 2 = retired). ß = standardized regression coefficient using “estadd beta; matrix list e(beta)” (not available in Stata when interactions are included).

##### Moderation of depression and anxiety symptoms and pre-/post-pandemic declaration

###### Depression symptoms

**FIGURE 3:**
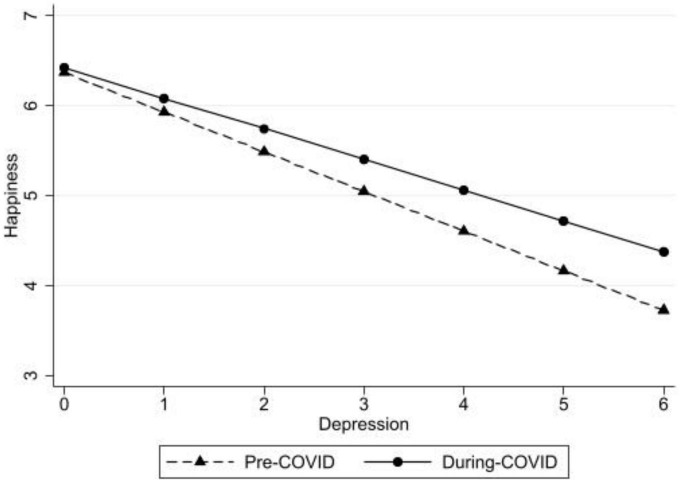
Depression symptoms predicting happiness pre- and post- the COVID-19 pandemic declaration with the follow-up sample.

To address hypothesis 3 if taking the survey pre- or during the COVID-19 pandemic for follow-up participants moderated the depression symptoms to happiness link among our sample, interaction effects were added to the multilevel models (see Model 2 of Table 4). Significant interaction effects were found between depression symptoms and survey time participation (pre-/post- pandemic declaration: *b* = 0.16, *p* = 0.001; see Figure 3 for a graphic representation of depression symptoms by pre-/post-pandemic declaration in predicting happiness). A simple slopes test indicated that depression symptoms were significantly linked with happiness for both pre- and post-pandemic declaration responses.

###### Anxiety symptoms

**FIGURE 4:**
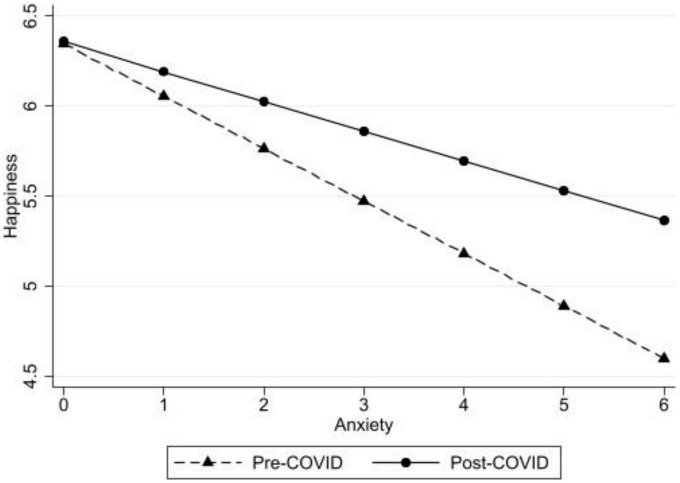
Anxiety symptoms predicting happiness pre- and post- the COVID-19 pandemic declaration with the follow-up sample.

To address hypothesis 3 if taking the survey pre- or during the COVID-19 pandemic for follow-up participants moderated the anxiety symptoms to happiness link among our sample, interaction effects were added to the multilevel models (see Model 2 of Table 4). Significant interaction effects were found between anxiety symptoms and survey time participation (pre-/post-pandemic declaration: *b* = 0.17, *p* < 0.000; see Figure 4 for a graphic representation of anxiety symptoms by pre-/post-pandemic declaration in predicting happiness). A simple slopes test indicated that anxiety symptoms was significantly linked with happiness for both pre- and post-pandemic declaration responses.

### Discussion

Adding to Study 1, we explored the longitudinal relationship between anxiety and depressive symptoms and happiness over the COVID-19 pandemic, using data collected from older adults residing in a Western state in the U.S. Findings matched Study 1’s results, which supported a resilience hypothesis. Specifically, higher levels of anxiety and depressive symptoms predicted lower levels of happiness; however, for those who indicated higher levels of anxiety and depressive symptoms, post-pandemic declaration happiness levels were higher than pre-pandemic happiness levels. This suggests that older adults have demonstrated resilience, to some extent, when they experienced higher levels of anxiety and depressive symptoms prior to the pandemic.

In support of hypotheses 1a and 1b, higher levels of anxiety and depressive symptoms were related to lower levels of happiness. Consistently, mental health symptoms have been associated with lower levels of happiness and life satisfaction, even among older adults (Fakouri and Lyon, 2005; Luchesi et al., 2018). Further, this relationship has been shown to last across time, with depressive symptoms predicting lower life satisfaction 6 years later (Berg et al., 2009). Because happiness is defined as the presence of positive emotions and the absence of negative emotions (i.e., hedonia; Ryan and Deci, 2001), findings from the current study suggests that negative emotions derived from anxiety and depressive symptoms are associated with lower levels of happiness.

Contrary to hypothesis 2a (i.e., stress-pile up theory), but in partial support of hypothesis 2b, the COVID-19 pandemic was related to higher levels of happiness. Such results may suggest a resilience framework, as older adults did not just remain stable in their reports of happiness, but in actuality fared better in their reports of well-being in spite of a worldwide pandemic.

Potential explanations of this finding relate to unique aspects of later life that could help with the development of resilience. From a biological perspective, previous research has found that positive behavioral changes parallel functional imaging studies, showing diminished responsiveness of amygdala to negative or stressful images in older adults (Mather et al., 2004). Such positivity may lead to higher levels of subjective well-being in later life. Other research suggests that older adults are better about letting go of past failures and pay more attention to what makes them happy presently (Klausen, 2020; Bauger et al., 2021; Casali et al., 2021). Perhaps such developmental changes act as an adaptive resource that has benefited older adults during the COVID-19 pandemic.

From a life course perspective, older adults have ample experience with stressful situations and have developed adaptive coping skills over time to adjust to novel events, like the COVID-19 pandemic (MacLeod et al., 2016). A 2020 Stanford University survey found that aging is associated with greater emotional well-being, even in the face of prolonged stress (De Witte, 2020). Older adults often demonstrate resilience by focusing on optimism, hopefulness, gratitude, and happiness, suggesting that when faced with difficulty, they will cope by directing their attention to maintaining positive emotions (MacLeod et al., 2016) and focusing on long-term utility rather than immediate gains (Worthy et al., 2011). Further, researchers have reported that, compared to younger adults, older individuals tend to be more skilled at emotional regulation, complex social decision making, information processing (Blazer, 2006; Worthy et al., 2011), and showing positive biases in their memory (Reed and Carstensen, 2012). All of these may contribute to the resilience demonstrated by many older adults. As such, recently published research has found that older adults seem to be handling pandemic stress much better than younger individuals (Birditt et al., 2021). In fact, another recent survey of 9,000 adults in the US and Canada across five generations (Lem, 2020; Jones, 2021) found that older respondents reported the highest percentages of coping “very well” with the pandemic (Birnstengal, 2020).

Another explanation for our finding may be a result of the increased time available to engage in physical activity. Research has consistently shown that exercise has a strong positive impact on mental health, including measures of well-being, less anxiety and depression symptoms, and emotional and stress resilience (Bernstein and McNally, 2018; Ashdown-Franks et al., 2019). Further, researchers have found that a negative change in exercise behavior before the COVID-19 restrictions was linked to poorer mental health and well-being, while those with more positive exercise behaviors indicating better mental health and well-being (Faulkner et al., 2021). As such, it may be that during the pandemic, individuals had increased time alone and so were able to exercise more thus impacting happiness levels. In addition to exercise, it may be that older adults enjoyed increased time spent outdoors. “Green exercise,” or any activity that takes place in nature, provides both physical and mental health benefits (Barton and Pretty, 2010), possibly accounting for the increased in happiness observed in our study.

A final explanation for our results may be that older adults enjoyed positive benefits from technological engagements brought about by the COVID-19 pandemic. One of the biggest concerns of the pandemic was the increase in loneliness that many individuals felt (Krendl and Perry, 2021). To combat the loneliness derived from cities shutting down and quarantines being in effect, individuals turned to technology for social connections with others. With older adults being one of the most at-risk groups for negative outcomes of the Coronavirus (Center for Disease Control [CDC], 2021) and many experiencing a deep fear of infection (e.g., Federico et al., 2021), family members and friends of older individuals may have become more aware of their need for social connection (Sarwar et al., 2020), and may have taken opportunities to reach out to them in diverse ways during that time. Thus, social interactions through technology may have had a positive influence on the happiness levels of older adults during the pandemic (see Colenda et al., 2020).

There are a number of potential explanations for why happiness increased over the COVID-19 pandemic that could be explored in future research. It may be that there were benefits to slowing down, getting better or more sufficient rest/sleep, receiving community support, or being able to regulate social activity preferences (more contact with family members/close relationships, or less pressure for anxiety inducing social situations). In the current study, having a partner, a higher education degree, and sufficient income were found to contribute to resiliency and subjective well-being among older adults. These characteristics were not the focus of this study, but further research on increased happiness over the pandemic in correlation with relationship, educational attainment and financial security in older adults would contribute to researchers’ and policymakers’ understanding to enhance well-being in older adults. Whatever the explanations may be, we have found some silver linings amidst the COVID-19 challenges for older adults.

Contrary to hypothesis 3, our findings did not support a stress pile-up theory. Instead, taking into consideration the COVID-19 pandemic, our findings supported a resilience perspective. Higher depression and anxiety symptoms were indeed associated with lower levels of happiness. Surprisingly, post-pandemic declaration happiness levels were higher than pre-pandemic happiness levels. Below we discuss possible mechanisms to explain this resilience finding in relation to anxiety and depressive symptoms.

Older adults have been found to be resilient in many situations, calling upon a lifetime of experience and perspective to help them through difficult times. Individuals who experience mental health symptoms throughout their life may acquire adaptive processes that can enable them to live resilient lives (Ungar and Theron, 2020). Although in some instances mental health challenges may exacerbate distress during a pandemic (Losada-Baltar et al., 2021), resilience that comes from experience may act as a buffer to stressful circumstances. Research shows that many older adults have positively adapted their behaviors throughout the pandemic. They reached out to family and friends, pursued hobbies, exercised, and participated in faith communities as they tried to stay healthy and safe (Graham, 2020). Further, the increased awareness by others may have accompanied increased social support. Social support is known to be a protective factor against mental health symptoms (Li et al., 2021).

In contrast to greater socialization leading to greater happiness, those with mental health challenges may have benefited from feeling less pressure to socialize. Depression or anxiety symptoms may be a result of feeling pressured to follow the cultural social norm to be with people (Dejonckheere et al., 2017) or to portray an active or “perfect” lifestyle (Thompson et al., 2020). As the country shut down, and many Americans were forced to halt their lives, older adults who experience mental health symptoms may have felt relieved from the expectation to conform to social pressures. Of note, the current study did not measure pandemic-related anxiety (but rather, measured anxiety, and the study took place during the pandemic), and as such, the presence of this specific form of stress was not captured. Further, as other pandemic-related variables were not specifically assessed in the larger study, we know less about the exact effect of the COVID-19 pandemic on the anxiety and depressive symptoms for our sample. For example, one main pandemic stressor, namely the fear of infection, has recently been studied and researchers have found that such a fear has resulted in a change in the way we look at other individuals, thus altering our social interactions daily (Federico et al., 2021). As older adults were considered a vulnerable population during the COVID-19 pandemic, results may differ if examining pandemic-related variables specifically.

## Limitations and future directions

There are a number of limitations to the current study. First, our analytic sample was taken from a larger research initiative that was planned and started prior to the onset of the COVID-19 pandemic. Because of that, results should be interpreted with caution, as the pandemic was not a planned piece of the broader project. Second, our sample size of older adults who struggled during the pandemic was non-probabilistic (convenience sample) and modest in size, and thus may not have represented all groups of older adults. It is likely that older adults with easy access to the internet were more likely to respond to the survey. Future research could explore the same associations as this study in different groups of older adults to confirm replication of findings. Third, resilience was not measured as a separate variable in our survey but was identified as a pattern of associations in reports by older adults in the study. Family functioning, social participation, and trust in healthcare institutions may also play a role in resilience. Future research could examine how these potential protective factors buffer the impacts of risk factors during the current COVID-19 crisis. Fourth, although we captured anxiety and depression using an established short measure, these self-reported mental health symptoms did not specifically capture “pandemic stress.” Stress as a result of the pandemic itself should be further explored as the COVID-19 pandemic still affects many vulnerable populations.

Fifth, the current research was conducted among a community sample. Happiness among older adults in unique populations, such as clinical populations, those residing in long-term care, and those living in different countries, may have been impacted differently by the COVID-19 pandemic. Understanding happiness among those select groups may be an important factor to explore in future research. Last, due to limited space in the larger research project and in order to avoid participant fatigue with lengthy survey question, happiness in the survey was measured with a single item. We recognize this methodological limitation as we interpret results with caution. Future research could assess a more complex and complete picture of happiness among older adults around impactful events, and could explore challenges experienced by older adults who contracted COVID, who cared for family members with COVID.

## Conclusion

In conclusion, despite the challenges people have experienced as a result of the COVID-19 pandemic, some may have experienced positive benefits, and even resilience, during this time, thus providing a silver lining on what has been an otherwise difficult time for most of the world. Notably, results from this study show that some older adults with self-disclosed anxiety and depressive symptoms demonstrated resilience through greater happiness. Such resilience may have stemmed from inoculation of prior stressors, increased access to technology and social support, or other varying resources. Perhaps social expectations were lessened during the pandemic, allowing those with anxiety and depression to feel happier and less pressured during the pandemic. Findings from this study suggest that resilience may be learned throughout a lifetime, and that experiences from prior stressors may show benefits in responding to future ones. Further, resilience may occur in vulnerable populations such as those with anxiety and depressive symptoms, and those in their later years. With greater knowledge and perspective, older adults may experience less distress and more hope when enduring future natural disasters and crises.

## Data availability statement

The datasets presented in this article are not readily available because it was not stated in the IRB that data would be open for public use. Requests to access the datasets should be directed to MS, melanie.serrao.hill@gmail.com.

## Ethics statement

The studies involving human participants were reviewed and approved by Brigham Young University IRB. The patients/participants provided their written informed consent to participate in this study. Written informed consent was obtained from the individual(s) for the publication of any potentially identifiable images or data included in this article.

## Author contributions

MS: conceptualization, investigation, methodology, formal analysis, data curation, writing—original draft, writing—review and editing, visualization, supervision, and project administration. NH: conceptualization, investigation, writing—original draft, writing—review and editing, visualization, project administration, and funding acquisition. JY: conceptualization, methodology, formal analysis, writing—original draft, writing—review and editing, visualization, supervision, and project administration. CB and KT: conceptualization and writing—original draft. All authors contributed to the article and approved the submitted version.
